# Perioperative management and outcomes of hip and knee arthroplasty among heartmate 3 left ventricular assist device recipients

**DOI:** 10.1007/s00590-026-04692-3

**Published:** 2026-03-31

**Authors:** Oguz Turan, Ignacio Pasqualini, Khaled A. Elmenawi, Shujaa T. Khan, Raya Nahlawi, Paulino Alvarez, Nicolas S. Piuzzi

**Affiliations:** 1 Department of Orthopaedic Surgery, Cleveland Clinic, Cleveland, USA; 2 Case Western Reserve University, Cleveland, USA; 3 Division of Cardiology, Cleveland Clinic, Cleveland, USA

**Keywords:** HeartMate 3, Left ventricular assist device (LVAD), Hip and knee arthroplasty, Perioperative management, Postoperative outcomes

## Abstract

**Purpose:**

We aimed to evaluate preoperative cardiovascular assessment, perioperative management, and outcomes in HM3 recipients undergoing hip or knee arthroplasty.

**Methods:**

This retrospective case series included six patients with HM3 LVADs undergoing total hip or knee arthroplasty. Mean age was 67.4 ± 5.7 years; two were female, and two had ischemic cardiomyopathy. Average ejection fraction was 15 ± 4.5%.

**Results:**

Procedures included three knee arthroplasties (50%), two total hip arthroplasties (33.3%), and one hip hemiarthroplasty (16.7%). Median time from LVAD implantation to arthroplasty was 19.2 ± 13.6 months. Patients were admitted an average of 2.7 ± 1.8 days preoperatively; mean postoperative length of stay was 13 days. All patients were anticoagulated with warfarin (mean INR 1.4 ± 0.2), with five requiring heparin bridging. LVAD-related infections developed in three patients (50%), and two (33%) required readmission. At final follow-up, two patients (33%) had died, at a mean of 28 ± 8 months post-arthroplasty and 47 ± 36 months after HM3 implantation.

**Conclusion:**

Hip and knee arthroplasty in HM3 LVAD recipients can be performed with perioperative management strategies that mitigate excess mortality beyond expected LVAD outcomes. These findings highlight the importance of specialized perioperative care in improving the safety and feasibility of arthroplasty in HM3 LVAD patients. Larger studies with longer follow-up are needed to refine patient selection, optimize perioperative care, and clarify the value of arthroplasty in this complex population.

## Introduction

With an estimated 62,000 Americans eligible for left ventricular assist devices (LVADs) due to advanced heart failure (HF), the patient population with this unique treatment device continues to expand [1, 2]. Implantable either as a bridge therapy for heart transplant or a destination therapy for those unable to get an orthotopic open heart transplant, over 2000 LVADs are implanted yearly in the United States [3–6]. Recent work has demonstrated that LVADs used as destination therapy has potentially increased survival over the long term in these patients to the point where elective surgeries are possible [3, 7–12]. Due to the increasing prevalence of LVAD patients as well as their increased survival, more patients are presenting to orthopaedic surgeons for elective surgeries such as total hip (THA) and knee arthroplasties (TKA) [13–15]. In addition to quality of life improvements for patients, total joint arthroplasties can lead to improvements in cardiovascular function and fitness especially with the decreasing rates of mortality for even patients with multiple comorbidities [16–20]. However, the risks of these procedures remain relatively unquantified due to the sparsity of the medical literature with few case reports and one case series [21–23]. Moreover, these reports lack information about perioperative management and the type of left ventricular assist devices. As the patients with LVADs undergo significant changes in physiologic and pharmacological parameters, the staff should be familiar with the dynamics associated with these devices [24]. Indeed, the existing scarce literature evaluates patients on older generation LVADs that have been removed from the market or discontinued, limiting generalizability. In the most recent manuscript, Rosenberg et al. [21] described a series of five patients with twelve surgeries and found three of the patients survived to receive a transplant while two died from strokes 16 months and 20 months after the index arthroplasty. However, no distinction was made between the types of LVADs used in the patients in their study [21]. Due to the Class I recall of the HeartWare Ventricular Assist Device (HVAD) and the discontinuation of Heartmate 2, the only current possibility is the Heartmate 3 (HM3) [25, 26]. As the risks of mortality, stroke, and complications changes drastically per LVAD type, this indicates a shortcoming in the predictability of outcomes for these patients [14]. Furthermore, all patients undergoing durable LVADs currently will all receive the Heartmate 3, making the requirement for further studies much more pertinent. With less than ten patients with LVADs receiving arthroplasty reported in the literature, the information for orthopaedic surgeons remains sparse regarding this challenging population. Furthermore, the great majority if not all patients in the literature have received LVADs that are no longer on the market, making their data far less relevant to clinicians. Subsequently, this study aims to analyze a larger cohort of LVAD patients undergoing total joint arthroplasty after having received the Heartmate 3 to better characterize the perioperative risks and complications in this unique population. Specifically, this study aimed to evaluate (1) pre-operative cardiovascular evaluation, (2) perioperative management, and (3) outcomes among Heartmate 3 (HM3) LVAD recipients undergoing hip or knee arthroplasty.

## Methods

### Study design and setting

This is a retrospective, observational study that leveraged a longitudinally maintained institutional database. We queried for patients who had both THA/TKA utilizing current procedure and terminology (CPT) codes, as well as an LVAD prior to the surgery. In order to capture patients who had LVADs from prior institutions, we utilized CPT Z95.811 for an additional search. A consecutive series of patients with LVADs were identified, who subsequently underwent hip or knee surgery at a large tertiary academic center in the Midwestern United States between October 2015 and June 2024. For these patients, demographic, clinical, imaging, pharmacologic, and hemodynamic data was reviewed. This study interval was chosen since the first HM3 implantation at our institution was in October 2015 in clinical trials prior to the approval of the HM3 LVAD by the FDA. The patients identified as having received arthroplasties after LVAD implantation were then manually reviewed for whether their LVADs were Heartmate IIIs prior to inclusion. Patient perioperative right heart catheterization and echocardiogram were looked at and data extracted if they were less than four months prior to the operation. Patients who received temporary LVADs as well as implants not currently on the market were excluded from inclusion. Analyses were performed using R software (R Core Team 2023), version 4.2.3. Variables were described using means ± standard deviations.

## Ethical considerations

This study was approved by the ethics committee of our institution.

## Results

### Study population

Initial search found 24 patients who had received any kind of LVADs prior to surgery, of whom 5 had older LVADs not currently on the market (i.e., HM2 or HVAD) while 13 had received temporary LVADs (i.e., Impella, TandemHeart). Our final cohort consisted of 6 patients with HM3 LVADs who underwent either THA or TKA at our institution between the study period. The mean age at LVAD implantation was 66 ± 6 years (range 53–72 years). The mean age at the time of arthroplasty was 67.4 ± 5.7 years (range 56–74 years). Four patients (67%) were male, and 2 patients (33%) were female. All six patients were white (100%). The mean body mass index at arthroplasty was 31.8 ± 5 kg/m^2^ (range 28–37 kg/m^2^). The mean follow-up time after arthroplasty was 39.5 ± 23.3 months (range 24–76 months). The mean Charlson Comorbidity Index was 7 ± 2 (range 5–11). All patients were managed perioperatively by a multidisciplinary team including cardiology and orthopedic surgery using standardized protocols. Of our patients, four (67) underwent right heart catheterizations less than four months prior to the operation while all six underwent echocardiograms within four months prior to the operation.

## Patient characteristics

Among the 6 LVAD patients undergoing arthroplasty, 2 (33%) were primary total hip arthroplasties, 2 (33%) primary total knee arthroplasties, 1 (17%) revision total knee arthroplasty, and 1 (17%) hip hemiarthroplasty. Primary osteoarthritis was the most common indication in four patients (67%), with one (17%) femoral neck fracture, and one patient (17%) who underwent a staged revision knee arthroplasty for prosthetic joint infection, with the initial explant/debridement surgery and temporary spacer placement followed 15 weeks later by re-implantation of the final knee components (Table 1).

**Table 1:** Baseline demographics and LVAD data

Variable	Level	Value
Age, mean ± SD (range)	At time of LVAD	66 ± 6 (53–72)
	At time of hip/knee surgery	67.4 ± 5.7 (56–74)
Sex, n (%)	Male	4 (67%)
	Female	2 (33%)
Race	White	6 (100%)
Smoking Status	Never	1 (17%)
	Former	5 (83%)
	Current	0 (0%)
BMI, mean ± SD (range)		31.8 ± 5 (28–37) kg/m^2^
Insurance	Medicare	5 (83%)
	Private	1 (17%)
CCI, mean ± SD (range)		7 ± 2 (5–11)
Initial therapy	Destination	5 (83%)
	Bridge to transplant	1 (17%)
Preoperative labs	Hemoglobin	11.3 ± 2.6
	Creatinine	1.28 ± 0.62
	EGFR	59 ± 23
	Platelets	170 ± 108
Comorbidities	Chronic kidney disease	3
	Diabetes mellitus	3
	Hypertension	2
	Malignancy	2
	COPD	2
	Thromboembolic event (PE/DVT/stroke)	2

Of the six patients, 1 (17%) received an LVAD for bridge to transplant while 5 (83%) had a destination LVAD. The etiology of heart failure was ischemic cardiomyopathy in two (33%) and non-ischemic cardiomyopathy in four (67%) patients. Notable preoperative labs consisted of an average hemoglobin of 11.7 ± 2.7, creatinine 1.26 ± 0.57, EGFR 61 ± 25.9, and Platelets 203 ± 149.

Regarding their cardiac demographics, the average cardiac index closest to surgery was 1.63 ± 0.12 L/min/m^2^, average cardiac output closest to surgery was 5.05 ± 1.56 L/min, and an average ejection fraction of 15 ± 4.5 (10–20) %. All our patients had some degree of mitral regurgitation with 1 (17%) having severe, 2 (33%) having moderate, and 3 (50%) having mild regurgitation. The left ventricular inner diameters during diastole and systole were 6.6 ± 1.02 (4.8–7.7) cm and 5.98 ± 1.08 (4.3–7.2) cm respectively. Considering the right ventricle, half (3) of our patients had moderately decreased function with 2 (33%) having mildly decreased and one patient having normal RV function. Right atrial pressures were 9.5 ± 4.7 (6–16) mmHg and right ventricle pressures were 33 ± 5.0/ 9.5 ± 4.8 mmHg. One patient (17%) had trace tricuspid regurgitation while two patients each had mild (33%) and moderate (33%) with the last patient (17%) having severe regurgitation. Furthermore, all six of our patients had a history of atrial fibrillation while two (33%) had an implantable cardioverter defibrillator (ICD).

### In-hospital metrics

The mean preoperative hospital stay was 2.7 ± 1.8 days (range 1–6 days). Using the American Society of Anesthesiologists (ASA) classification, all 6 patients were ASA class 4. All 6 patients underwent general endotracheal anesthesia. The mean operative time was 125 ± 59 min (range 51–179 min) and the mean estimated blood loss was 450 ± 480 mL (range 100–1500 mL). Two patients required blood transfusions (2 and 5 units of packed red blood cells). There were no intraoperative or immediate postoperative complications. The mean hospital length of stay was 13 ± 5 days (range 8–26 days). Three patients (50%) were discharged home while the other 3 (50%) were discharged to acute care facilities. Regarding operative and management parameters, all patients had an LVAD specialist team in the operating room along with a cardiothoracic surgery representative. Of the six patients, five (83%) underwent anesthesia using a cardiothoracic fellowship trained anesthesiologist. (Table 2) Preoperatively, all patients were anticoagulated on warfarin. Five patients (83%) received a heparin bridge prior to surgery, with a mean time from heparin initiation to surgery of 2 ± 1 days (range 0–3 days). (Table 3**).** The INR closest to surgery was 1.4 ± 0.2 (range 1.2–1.7) with a baseline INR of 2.2 ± 0.5 (1.4–2.9). The mean preoperative PTT was 48 ± 11 s (range 26–60 s). Intravenous heparin was restarted on postoperative day (POD) 0 for one patient, POD 1 for 3 patients, and POD 2 for 1 patient and POD3 for the last patient. One patient received aspirin postoperatively while five patients were on warfarin, with a mean time to restart anticoagulation of 36 ± 15 h (range 10–52 h).

**Table 2:** In-hospital metrics

Variable	Level	Value
Time from admission to arthroplasty	In days	2.7 ± 1.8 (1–6)
ASA classification	4	6 (100%)
Anesthesia	GETA	6 (100%)
Anesthesiologist fellowship	Cardiothoracic anesthesia	5 (83%)
	Critical care	1 (17%)
Operative time	In minutes	125 ± 59 (51–179)
EBL	In mL	450 ± 480 (100–1500)
Transfusion of blood products	0 U PRBC	4 (66%)
	1 U PRBC	0 (0%)
	2 or more U PRBC	2 (34%)
Complications	Intra-operative	0 (0%)
	In-hospital	0 (0%)
Length of stay	In days	13 ± 5 (8–26)
Discharge disposition	Home +/- Home Health Care	3 (50%)
	Acute Care Facility	3 (50%)
Joint	Hip	3 (50%)
	Knee	3 (50%)
Procedure type	TKA	3 (50%)
	THA	2 (33%)
	Hip hemiarthroplasty	1 (17%)
Surgical indication	Primary osteoarthritis	4 (66%)
	Femoral neck fracture	1 (17%)
	Prosthetic joint infection	1 (17%)

**Table 3:** Cardiovascular background

Variable	Level	Value
Cardiac index	Closest to surgery	1.63 ± 0.12 L/min/m^2^
Cardiac output	Right heart cath (*n* = 4)	5.05 ± 1.56 L/min
Diagnosis of HF	Ischemic cardiomyopathy	2 (33%)
	Non-ischemic cardiomyopathy	4 (67%)
ICD	Yes	2 (33%)
	No	4 (67%)
Atrial fibrillation	Yes	6 (100%)
	No	0 (0%)
Medications	Aspirin	4
	Entresto	1
	Beta-blocker	3
	Loop diuretic	5
	Aldosterone agonist	4
	Digoxin	2
	Amiodarone	3
Ejection fraction	Preoperative echo	15 ± 4.5 (10–20)%
Left ventricle inner diameter	Diastole	6.6 ± 1.02 (4.8–7.7)
	Systole	5.98 ± 1.08 (4.3–7.2)
Right ventricle function	Normal	1 (17%)
	Mild decrease	2 (33%)
	Moderately decreased	3 (50%)
Right atrium pressure	Right heart cath (*n* = 4)	9.5 ± 4.7 (6–16) mmHg
Right ventricle pressure	Right heart cath (*n* = 4)	33 ± 5.0 / 9.5 ± 4.8 mm Hg
Pulmonary artery pressure	Right heart cath (*n* = 4)	33.5 ± 6.5 mm Hg
Pulmonary capillary wedge pressure (PCWP)	Right heart cath (*n* = 4)	17.8 ± 5.7
Mitral regurgitation	Mild (+ 1)	3 (50%)
	Moderate (2+-3+)	2 (33%)
	Severe (4+)	1 (17%)
Tricuspid regurgitation	Trace	1 (17%)
	Mild (+ 1)	2 (33%)
	Moderate (2+-3+)	2 (33%)
	Severe (4+)	1 (17%)
Pre-op anticoagulant	Xa Inhibitor	3 (50%)
	Warfarin	3 (50%)
Pre-op INR	Closest to surgery	1.4 ± 0.2 (1.2–1.7)
	Baseline	2.2 ± 0.5 (1.4–2.9)
Pre-op PTT	Closest to surgery	48 ± 11 (26–60)
IV heparin bridge	Yes	5 (83%)
	No	1 (17%)
Time from bridge to arthroplasty	In days	2 ± 1 (0–3)
Time to resume IV heparin	POD#0	1 (17%)
	POD#1	3 (50%)
	POD#2	1 (17%)
	POD#3	1 (17%)
Post-op anticoagulant	Warfarin	5 (83%)
	Aspirin	1 (17%)
Time to restart PO anticoagulation	In hours	36 ± 15 (10–52)

## Postoperative outcomes and complications

Early postoperative outcomes evidenced a 33% 30-day readmission rate - one patient for multiorgan failure leading to expiration and another for pneumonia. The average follow-up time after TJA ranged between 24 and 76 months, with an average of 39.5 months. At latest follow-up, one (17%) patient underwent revision of the hip/knee arthroplasty components. Out of the LVAD-related complications studied, including infection, hemorrhage, stroke, pump thrombosis, and death, only infection occurred in 3 patients (50%) - all driveline infections including one case of persistent Stenotrophomonas maltophilia progressing to fatal ARDS. At final follow-up (mean 39.5 ± 23.3 months), 4 patients (66%) were alive. The 2 deceased patients had an average survival of 28 ± 8 months after arthroplasty and 47 ± 36 months after HM3 implantation. Out of these two patients, the causes of death were ARDS from Stenotrophomonas within the immediate postoperative period complicated by septic shock and multiorgan failure. The other patient had a persistent dialysis catheter infection who remained febrile despite being on prophylactic meropenem and eventually elected to go to hospice instead. (Table 4)

**Table 4:** Patient outcomes

Variable	Level	Value
Any LVAD complication	Yes	3 (50%)
	No	3 (50%)
Type of LVAD complication	Infection	3 (100%)
	Hemorrhage	0 (0%)
	Stroke	0 (0%)
	Pump thrombosis	0 (0%)
	Death	0 (0%)
30-day inpatient readmission	Yes	2 (33%)
	No	4 (67%)
Complications within 30-days Post-op	Multi-organ failure	1 (25%)
	Pneumonia	1 (25%)
Follow-up time	In months	39.5 ± 23.3 (24–76)
Hip or knee re-operation	Yes	1 (17%)
	No	5 (83%)
Mortality at final follow-up	Yes	2 (33%)
	No	4 (67%)
Time to mortality	LVAD date	28 ± 8 months
	Hip/Knee surgery date	18 ± 17 months

## Discussion

With growing numbers of advanced heart failure patients being treated with LVADs, more of these patients are increasingly opting for elective hip and knee arthroplasties to address degenerative joint disease. In this retrospective case series, the characteristics and outcomes of patients with HM3 LVADs undergoing hip or knee arthroplasty were explored. To date, this analysis represents the only examination focused specifically on arthroplasty outcomes among recipients of this LVAD model. This case series’ findings suggest that meticulous perioperative care by a dedicated multidisciplinary team is necessary to improve the safety of arthroplasty in select HM3 LVAD patients. Additionally, specialized perioperative care for this patient cohort may decrease the risk of perioperative mortality or stroke risk compared to overall outcomes for this population. However, due to the small sample size of this case series, future prospective studies should investigate the optimal perioperative care to decrease the risk of complications in this patient group. Additionally, the procedures necessitated substantially prolonged hospitalization and health care resources exceeding that typical of arthroplasty patients. Notably, the improved functional status enabled by joint replacement allowed one destination therapy LVAD patient to qualify for orthotopic heart transplantation. While these initial results indicate arthroplasty is technically feasible in LVAD recipients, the poor longer-term prognosis underscores the need for further data clarifying appropriate patient selection criteria and defining the risk-benefit profile of these highly complex procedures in a medically vulnerable cohort.

Prior studies have examined arthroplasty outcomes in LVAD recipients, but none have specifically focused on the HM3 device. Rosenberg et al. [21] included 5 patients for a total of 12 surgeries including bilateral and revision surgeries as separate instances. However, the authors did not characterize by the type of LVAD the patients had received. Walton et al. [22] described a case of a THA after a Heartmate II who survived to heart transplant after functional improvement [22]. Furthermore, Leonard et al. [23] reported two patients undergoing TKA with one patient surviving to transplant and the other developed Staphylococcus Aureus bacteremia unrelated to the arthroplasty and passed away after eight months’ post arthroplasty. However, the two patients analyzed had also both received the Heartmate II, limiting generalizability. Previous trials have also compared the newer centrifugal-flow HM3 LVADs with the older axial-flow HM2 LVADs. The pivotal MOMENTUM 3 trial compared HM3 LVADs with HM2 LVADs in patients with advanced heart failure and evaluated the 2-year risk of disabling stroke and reoperation to replace a nonfunctioning device. They found that the HM3 group had lower risk of disabling stroke and reoperation for pump replacement [27]. Another observational study by Mehra et al. analyzed 5-year outcomes of patients from the MOMENTUM 3 trial who either received HM3 or HM2 LVADs. They found that patients with the HM3 LVADs had significantly lower rates of pump thrombosis, stroke, and bleeding compared to HM2 patients at 5-year follow up. This study also illustrated an increased likelihood of overall survival in the HM3 group at the 5-year mark [28]. However, the risk of bleeding after either LVAD device remains high due to the use of anticoagulants to prevent device thrombosis, and so patient-centered peri-operative care in LVAD patients undergoing TJA is necessary to decrease the risk of peri-operative hemorrhage. The superior hemocompatibility and improved outcomes of the HM3 compared to the HM2 LVAD can be explained by the differing mechanisms between the two devices. While the HM2 device is placed thoraco-abdominally, and is a continuous flow device, the HM3 device is placed intrapericardially, uses intrinsic pulsatile flow, and has wide blood flow pathways [27, 28]. Therefore, the HM3 device has lower blood friction and reduces the risk of blood clotting. As each LVAD device confers different risks related to design and clinical performance, analysis specific to the HeartMate 3 will facilitate appropriately informed surgical decision-making and perioperative planning for this growing patient population.

Overall, LVAD recipients are at high risk for complications, with prior studies showing rates of up to 40–50% for device malfunction, 25–40% for bleeding, and 13–29% for stroke [21]. In this LVAD case series, the most frequent complications were related to LVADs were infections in 3 out of 6 patients (50%). A quarter of our patients experienced readmissions due to complications. This aligns with prior literature showing high rates of device-related complications and readmissions in LVAD patients [29, 30]. Moreover, the 67% survival rate at follow-up in this study compares favorably with mortality rates reported in prior literature, which range from 52 to 86% for LVAD patients over similar time period [7, 20, 31–33]. This is substantially higher than the approximately 2% 1-year mortality seen in the general arthroplasty population [34, 35]. However, LVAD patients undergoing arthroplasty have significantly higher baseline medical complexity, with all patients in our study classified as ASA 4. The fact that the mortality rate in our study compares similarly with patients that have LVADs without arthroplasty suggests that arthroplasty may not independently increase 1-year mortality risk when performed in carefully selected patients. However, studies with longer follow-up and larger sample sizes are warranted, given the ongoing mortality hazard posed by the substantial medical complexity and comorbidities in LVAD recipients over time. Additional research should further investigate the impact of arthroplasty on functional outcomes and transplant candidacy in this population.

In contrast to the previous studies, only a minority of our patients (33%) needed blood transfusions while 8 out of the 12 surgeries in Rosenberg et al. [21] and all three patients reported by Walton et al. [22] and Leonard et al. [23] required transfusions. The balance between anticoagulation and stroke is a fine line for LVAD patients since the incidence of stroke and hemorrhage in LVAD patient is extremely high when compared to the overall population [36–38]. All patients in the present study were on warfarin anticoagulation and were admitted multiple days prior to arthroplasty for optimum bridging and anticoagulation as per institutional guidelines. Although these patients are hemodynamically challenging, the fact that only a third of our patients required transfusions indicates that this risk can be compensated for.

The substantially prolonged length of stay seen in this study, averaging 13 days compared to 1–2 days typical for arthroplasty, reflects the greater perioperative risks and delayed recovery in this medically complex population [21, 23, 39]. The innate heart failure and activity limitations of LVAD recipients likely contribute to slower gains in postoperative mobility and delays in meeting discharge criteria. However, the extended hospitalization may also be driven by the need for intense perioperative management by a multidisciplinary LVAD team to optimize anticoagulation, prevent device complications, and closely monitor for bleeding, thrombosis, arrhythmias, and other medical issues. While intrinsic factors related to the underlying heart failure play a role, the complexity of perioperative LVAD care itself also necessitates an abundance of caution and significantly longer hospitalization compared to routine arthroplasty patients. Further research could help delineate the relative contribution of patient comorbidities versus specialized perioperative management in dictating the prolonged lengths of stay.

LVADs play a critical role as either bridge therapy for transplantation or as destination therapy to prolong life in advanced heart failure patients. In this study, most patients (83%) received an LVAD for destination therapy while only 1 patient had a bridge device. This aligns with prior literature showing that most LVADs are implanted for destination therapy, with only a small proportion being converted to transplant eligibility. For example, Goldstein et al. [40] found that only 13.5% of LVAD patients originally deemed transplant-ineligible were reclassified as candidates within 2 years. In contrast, Rosenberg et al. [21] found a much higher rate of conversion to transplant after arthroplasty, with 3 of 5 (60%) patients improving enough to undergo heart transplantation. This suggests that for selected LVAD patients limited by arthritis, successful joint arthroplasty may restore function and allow them to meet transplant criteria. While destination therapy with LVADs confers a survival benefit, transplantation remains the optimal therapy for eligible patients. Our findings indicate that arthroplasty may enable a subset of destination therapy LVAD patients to improve their status and undergo definitive transplant treatment.

This study has limitations common to retrospective case series, including a small sample size that precludes definitive conclusions. However, with six patients, it represents the largest case series of LVAD patients undergoing arthroplasty. The study population lacks a comparator group of arthroplasty patients without LVADs. Given the greater comorbidity burden and perioperative complexity of LVAD patients, a matched control group would better isolate the incremental risks attributable to the LVAD itself. Finally, complications were not reported in relation to patient characteristics due to the small sample size and low event rate. As such, further research may be needed to clarify these associations. Despite these limitations, this study provides a baseline understanding of the feasibility and risks of arthroplasty in carefully selected LVAD patients. Going forward, larger multicenter studies with comparator groups and extended follow-up are needed to better quantify outcomes in this population [41].

## Conclusion

Hip and knee arthroplasty patients with HM3 LVAD can be safely performed utilizing a specialized, multidisciplinary team at a tertiary hospital. However, the procedures require substantially increased health care resources, with length of stay greatly exceeding that of routine arthroplasty patients. The final follow-up mortality of approximately 33% was comparable to overall LVAD survival, suggesting arthroplasty may not independently worsen short-term outcomes when proper patient selection and perioperative care is implemented. However, the high mortality indicates continued risks of arthroplasty in this population with an inherently high mortality rate. The resource intensiveness and poor longer-term survival underscore the need for larger studies to better elucidate the cost-benefit profile, appropriate patient selection criteria, and overall value of pursuing these technically feasible but highly complex procedures in LVAD recipients. Careful consideration of expected postoperative longevity and quality of life is warranted when evaluating LVAD patients for elective hip or knee arthroplasty.

## Data Availability

No datasets were generated or analysed during the current study.
